# Prevalence of Adverse Drug Reactions in CAD STEMI Patients Treated in the Cardiac Intensive Care Unit at the Public Hospital in Bandung, Indonesia

**DOI:** 10.3797/scipharm.ISP.2015.08

**Published:** 2016-02-14

**Authors:** Lia Amalia, Kusnandar Anggadireja, Toni M. Aprami, Vina Septiani

**Affiliations:** 1 School of Pharmacy, Institut Teknologi Bandung, Indonesia; 2 Faculty of Medicine, Universitas Padjajaran, Indonesia

**Keywords:** ADRs, CAD STEMI, Naranjo, Hartwig, Schumock-Thornton scales

## Abstract

Adverse drug reactions (ADRs) are associated with morbidity, mortality, and can contribute to increased healthcare costs. This study was conducted to identify the occurence, types, and management of ADRs, as well as analyze the causal relationship, severity, and preventability of ADRs. The study was observational analysis with concurrent data collection from patients with Coronary Artery Disease-ST segment Elevation Myocardial Infarction (CAD-STEMI) treated in the Cardiac Intensive Care Unit (CICU) at a hospital in Bandung Indonesia, during the period of December 2013 to March 2014. The occurence of identified ADRs was assessed using the probability scale of Naranjo, while the severity by the scale of Hartwig and their preventability was evaluated using the scale of Schumock-Thornton. 49 ADRs were identified in 29 patients. Organ systems most affected by the ADRs were the cardiovascular and body electrolyte, each accounting for 20.41%. The hematology and gastrointestinal systems each contributed 18.37% to ADR occurrences. The causal relationship was mostly classified as “probable,” accounting for 69.39%. With regard to severity, most ADRs were classified as “moderate” at level 3, contributing to 53.06% of the occurence. In terms of preventability, most of the ADRs fell into the “non-preventable” category (79.59%). The most widely applied ADRs management was administration of an antidote or other treatments (40.82%). Further analysis revealed that the average number of drug types and duration of hospitalization significantly affected the presence of ADRs. Taken together, most patients with CAD STEMI treated in the CICU of the studied hospital experienced non-preventable ADRs and were treated with antidote or other treatments.

## Introduction

An estimated number of 17.3 million of patients died from cardiovascular diseases in 2008, representing 30% of all deaths globally. Eighty percent of deaths from cardiovascular disease occurs in countries with low and middle incomes, and occur almost equally between male and female [1]. Coronary Artery Disease (CAD) is the most common cause of death from cardiovascular disease (45% of all cardiovascular disease), with 7,200,000 deaths/year, or 12 percent of all deaths worldwide [2]. In many developing countries, the CAD is the leading cause of death [3]. In Indonesia, the prevalence of cardiovascular disease in individuals over 15 years of age was 9.2% [4]. Based on basic health research data, the prevalence of acute coronary syndrome was 7.2% in 2007 and ST-segment Elevation Myocardial Infarction (STEMI) was a spectrum of acute coronary syndrome with the highest severity.

The complexity and intensity of care required by patients in intensive care, was associated with greater risk of harm. Drug is a type of therapy most commonly used and associated with adverse events, which particularly most frequent in the intensive care unit. Patients with critical illness are at high risk of experiencing adverse events related to the use of drugs [5].

WHO defined adverse drug reactions (ADRs) as a response to a drug which is noxious and unintended, occurs at normal doses used in human for prophylaxis, diagnosis, or therapy of disease or for modifying the physiological functions [6]. A number of studies demonstrated that the ADR was one the major problems in health service, not only related to morbidity and mortality but also associated with increased healthcare costs [7]. Increased costs especially occur when ADRs cause prolongation of the treatment [8].

The present study was aimed to identify the occurence, types, and management of ADRs in CAD STEMI patients. This study further evaluated causal relationship, severity and preventability of the ADRs. This study will provide information about the ADRs in the pertinent field, for the monitoring and prevention of the occurrence of the ADRs as well as providing educational materials for health professionals.

## Results and Discussion

### Patients Demographic Analysis

**Fig. 1:**
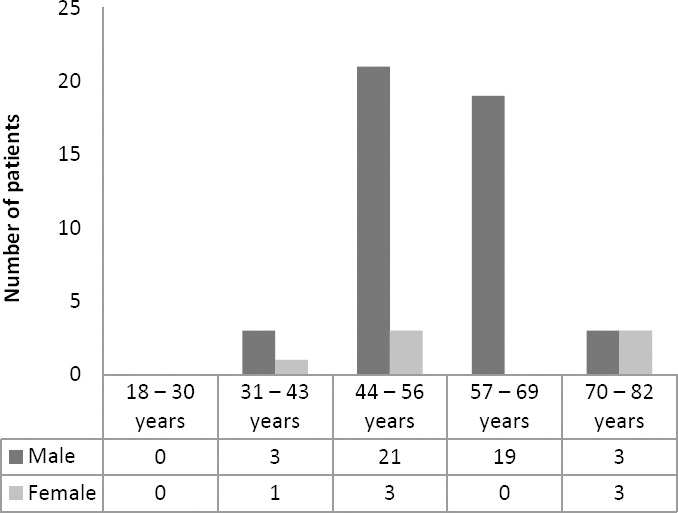
Distribution profile of CAD STEMI patients based on gender and age groups

**Fig. 2:**
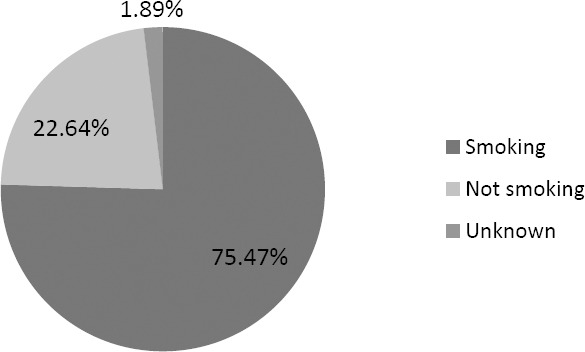
Profile of smoking history of CAD STEMI patients

As shown in Figure 1, 53 adult patients were diagnosed with CAD STEMI, the number of male were higher than female patients. The patients mostly fell into the range of age of 44–56 (24 patients, 45.28%). Figure 2 shows 75.47% of CAD-STEMI patients smoked, which portrays smoking as one risk factor in the onset of CAD. Indeed, some of the compounds in cigarettes, including nicotine and reactive aldehyde (such as acrolein), have been associated with endothelial dysfunction and atherosklerosis in smokers [9].

**Fig. 3:**
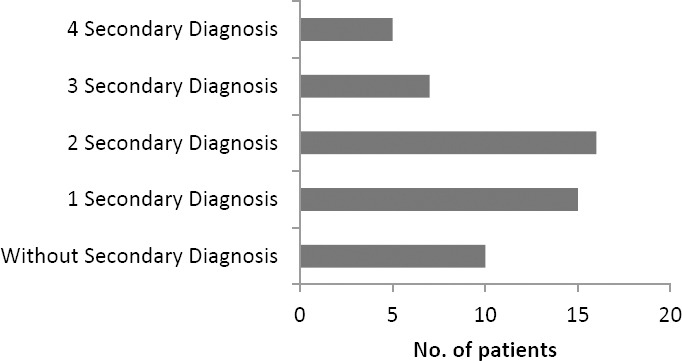
Profile of Distribution profile of patients with secondary diagnosis in CAD STEMI patients

**Fig. 4:**
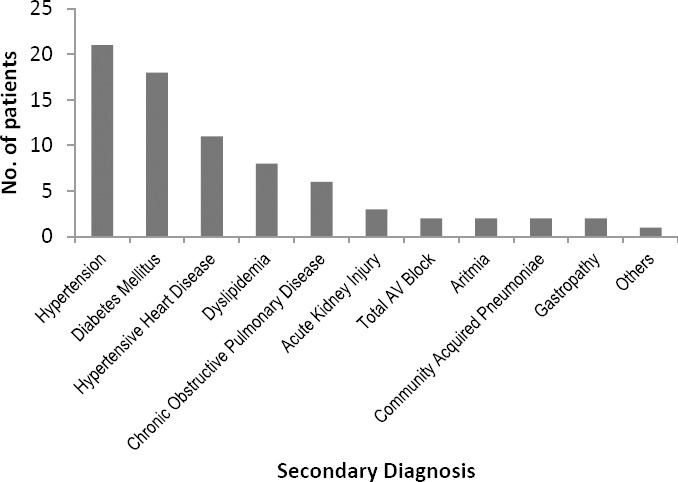
Profile of secondary diagnosis in CAD STEMI patients

Figure 3 and 4 present the number of patients with secondary diagnosis. Patients with secondary diagnosis tended to use more drugs, thus more potential ADRs were observed. The two most prevalent secondary diagnoses in CAD STEMI patients studied were hypertension and diabetes mellitus, two common risk factors for cardiovascular disease [10, 11].

### Analysis of Drugs Use

Table 1 and Table 2 present data on the drugs used by CAD STEMI patients. The drugs were classified into two groups, cardiovascular and non-cardiovascular drugs. Cardiovascular drugs included fibrinolytic, anticoagulant, antiplatelet, antihypertension, antihyperlipidemia, antiangina, diuretic, antiarrythmic and positive inotropic agents.

**Tab. 1:**
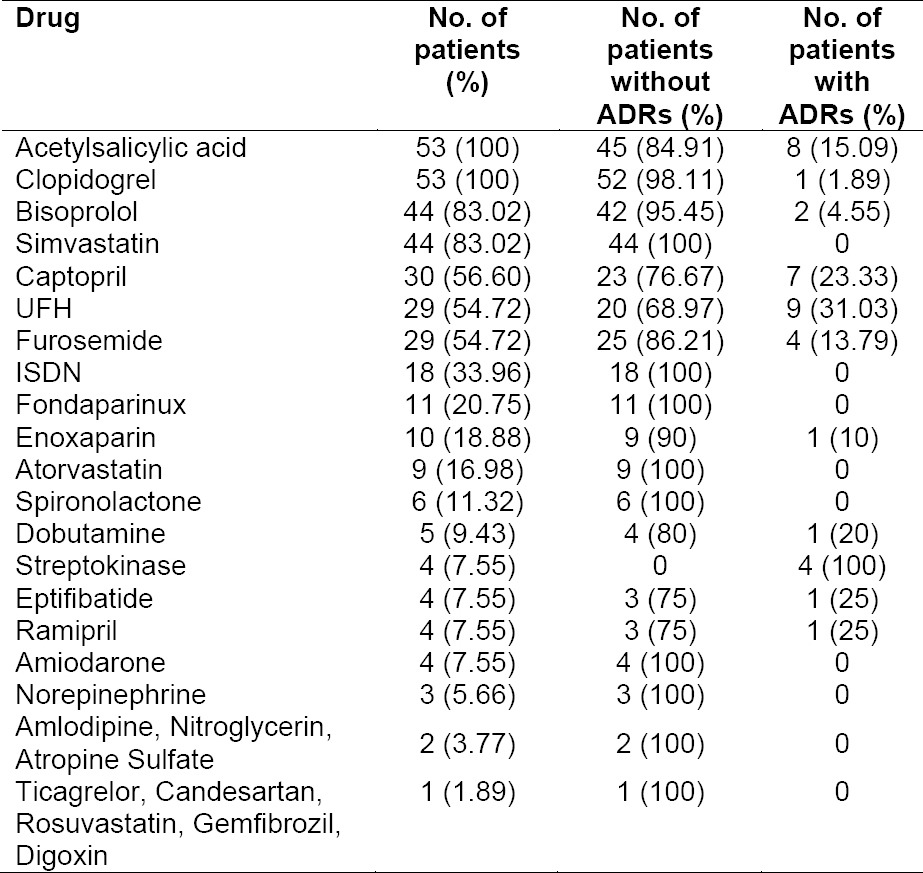
Cardiovascular drugs that used by and occurence of ADRs in CAD STEMI patients

**Tab. 2:**
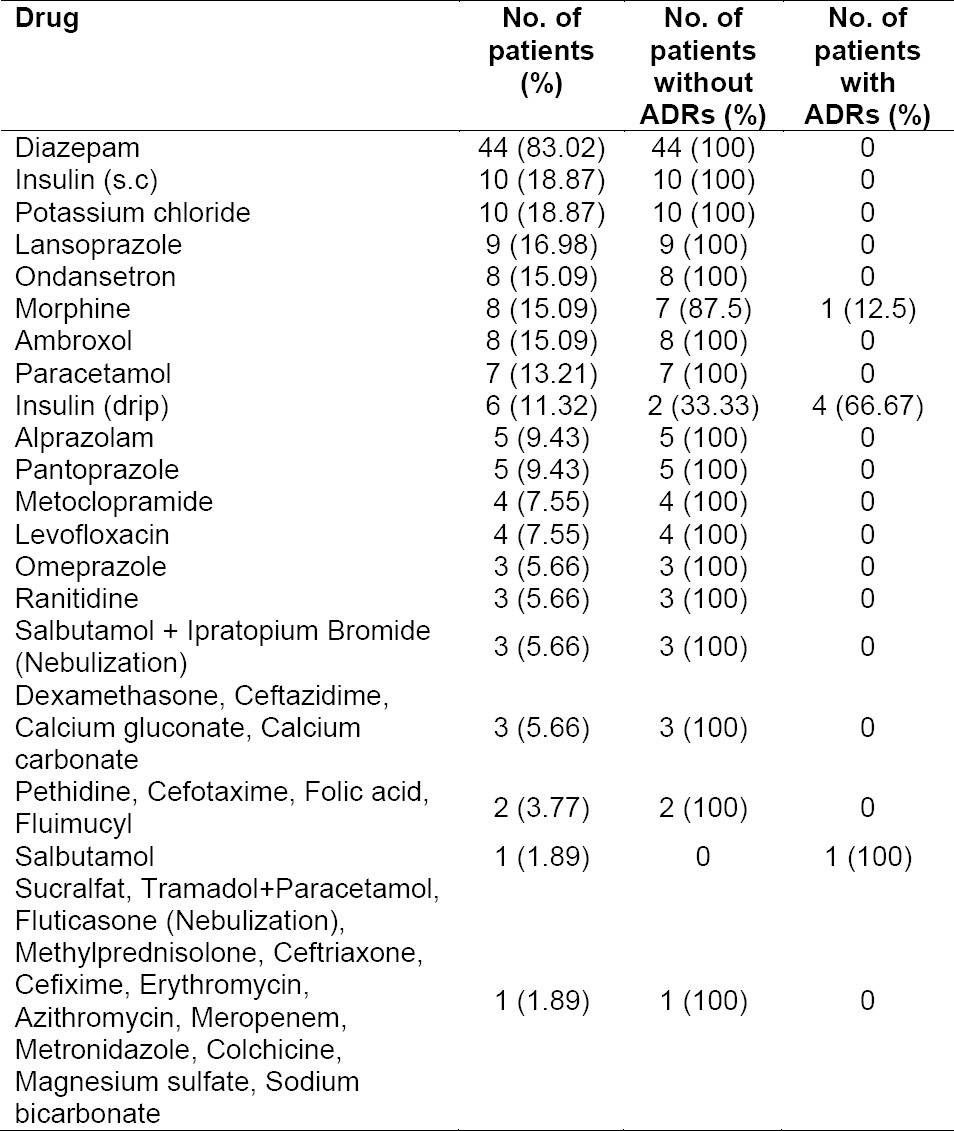
Non-cardiovascular drugs that used by and occurence of ADRs in CAD STEMI patients

The most widely used cardiovascular drugs were antiplatelet (acetylsalicylic acid and clopidogrel). All patients with CAD STEMI in this study used both drugs, known as Dual Antiplatelet therapy (DAPT). A clinical trial involving CAD STEMI patients showed that therapy with 75 mg clopidogrel once daily (started within 24 hours of admission to hospital) coadministered with acetylsalicylic acid for the duration of the treatment, reduced mortality and reinfarction occurence compared to placebo [12]. The second most used drugs were simvastatin and bisoprolol. Therapy with statins was shown to reduce long-term mortality in some subgroup of patients with CAD [13]. Simvastatin was the most widely used drug compared to other statins such as atorvastatin and rosuvastatin, possibly due to the availability of generic drugs and was included in the patient’s payment scheme. Bisoprolol, one of beta blockers, decrease myocardial ischemia and limits the infarct size. Early use of beta blockers in myocardial infarction has been shown to reduce the incidence of arrhythmias, decrease chest pain symptoms, and decrease sudden cardiac death and early and late re-infarction [14]. The most widely used non-cardiovascular drug was diazepam. Diazepam is used to lessen anxiety and helps patients to rest, which in turn could reduce the workload of the patient’s heart, supporting their recovery. Indeed, bed rest is one non-pharmacological therapy recommended in all patients with acute coronary syndrome [15].

### Adverse Drug Reactions Analysis

**Fig. 5:**
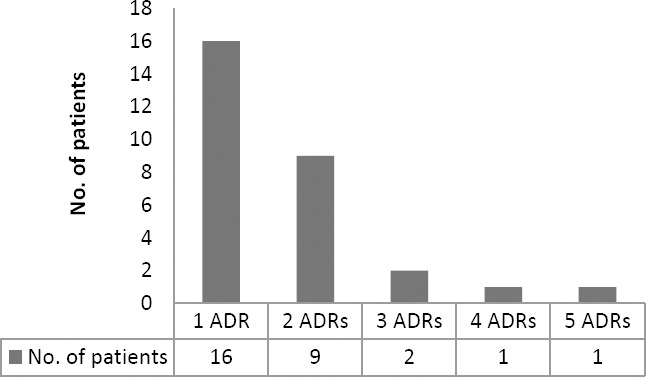
Profile of number of ADRs experienced by patients with CAD STEMI

As seen in Figure 5, 49 ADRs occurence were identified in 29 patients (54.72%), the detailed number of ADRs experienced by patients with CAD STEMI. One patient might have more than one ADR onset during treatment.

ADRs that occurred rendered the patient to get additional drugs for the management of the ADRs which could worsen the patient’s clinical condition. However the majority of patients (30.19%) only experienced one ADR. Table 3 indicates the organ systems affected in the occurrence of ADRs. The most affected systems were the cardiovascular system and body electrolyte, each accounting for 20.41%, with hypotension and hypokalemia as the most prevalent. Hypotension might be related with the use of streptokinase, captopril and bisoprolol. Other systems affected were hematological and the digestive systems, each contributing to 18.37% of the occurrence of ADRs. Falling into the hematological system, the increase in Activated Partial Thromboplastin Time (APTT) due to the use of unfractionated heparin (UFH) was the most frequent, whereas nausea, vomiting and heartburn were the most prevalent ADRs affecting the digestive system.

**Tab. 3:**
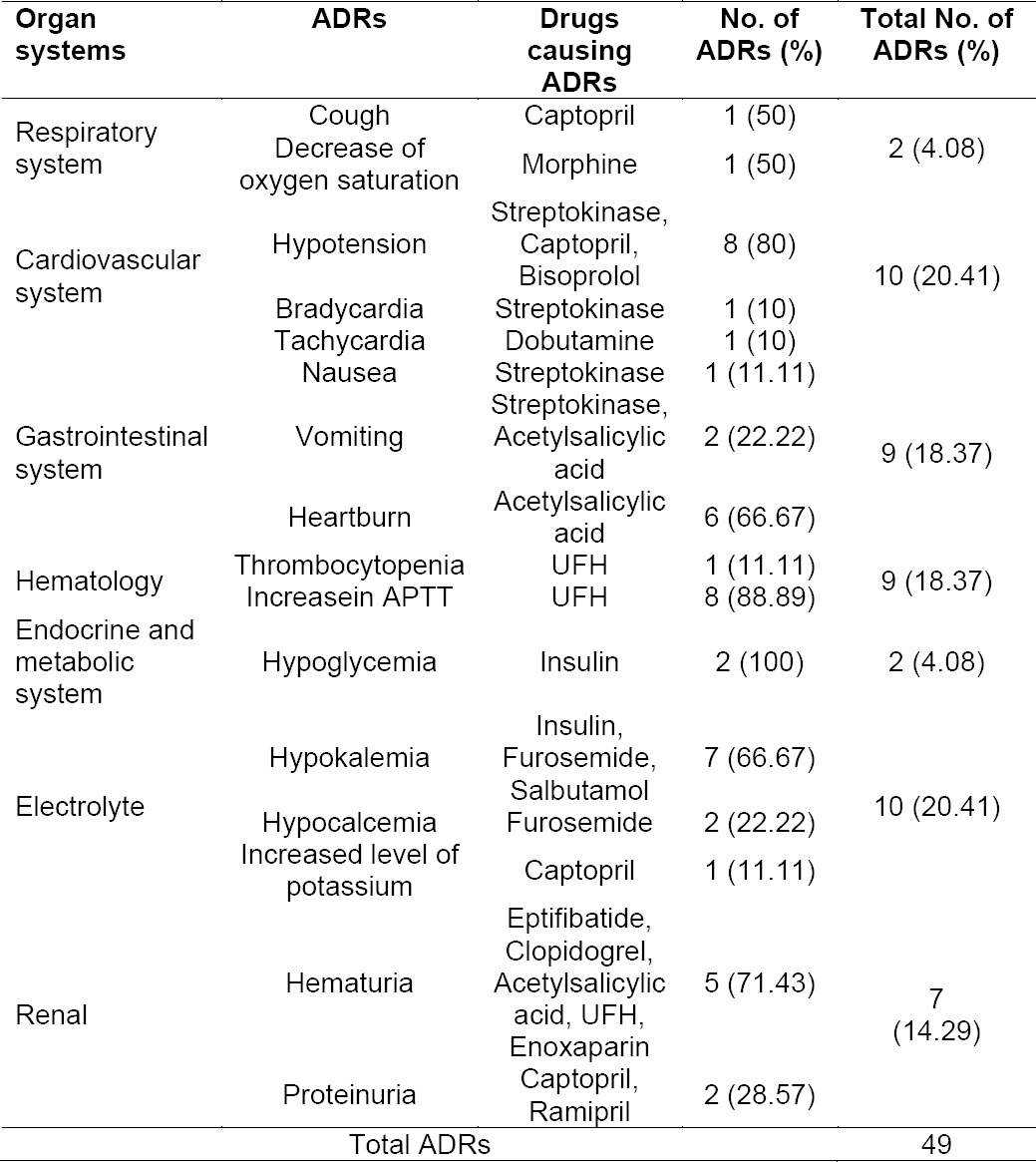
Profile of ADRs as related to organs systems affected and causing drugs

Tables 4, 5, and 6 show the data of ADRs occurrence in patients with CAD STEMI. As indicated, causal relation between drug and the occurrence of ADRs, mostly fell into “probable” category (34 events or 69.39%). ADRs were categorized “possible” due to the possibility of other causes besides drugs that induce such reactions. ADRs classified “certain” (definite), included hypotension, bradycardia and vomiting due to fibrinolytic therapy with streptokinase were clearly visible, because during dispensing, vital signs and patient’s condition were monitored continuously.

**Tab. 4:**
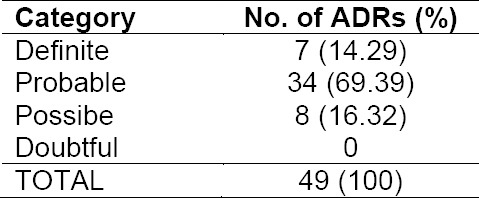
Result of causality assessment of identified ADRs

**Tab. 5:**
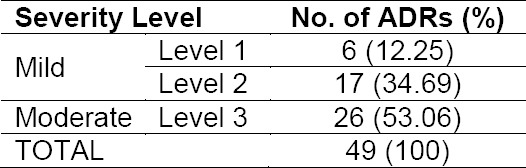
Result of severity assessment of identified ADRs

**Tab. 6:**
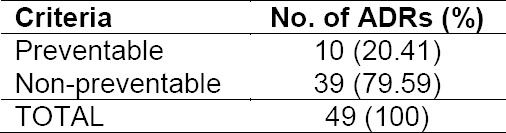
Result of preventability assessment of identified ADRs

The severity of the ADR level at most fell into level 3 or moderate (53.06%), the ADR that requires discontinuation, change and/or the use of an antidote or other treatments [16]. To assess the severity of the ADRs, the methods by which ADRs were managed should also be observed. The ADRs were mostly managed by administration an antidote or other treatments (Table 7). There were 34.69% of ADRs classified into level 2 severity. ADRs falling into this category need discontinuation of the suspected drug or modification, but does not require an antidote or other treatments, and no prolongation of hospitalization required. There were only six ADRs (12.25%) that belonged to level 1 severity. Although the ADRs with level 1 severity do not require the medication changing, the patient’s condition must be kept monitored.

**Tab. 7:**
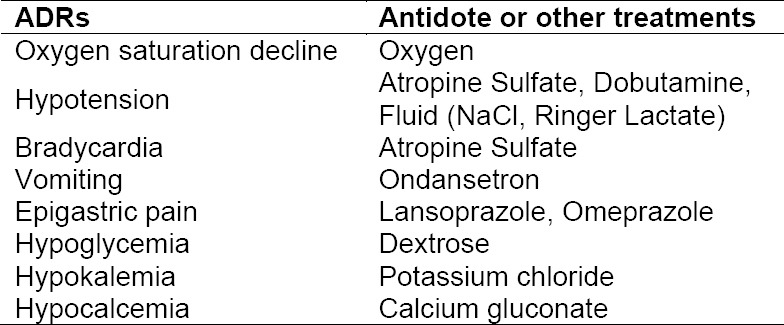
Antidote or other treatments in management of ADRs in CAD STEMI patients

**Tab. 8:**
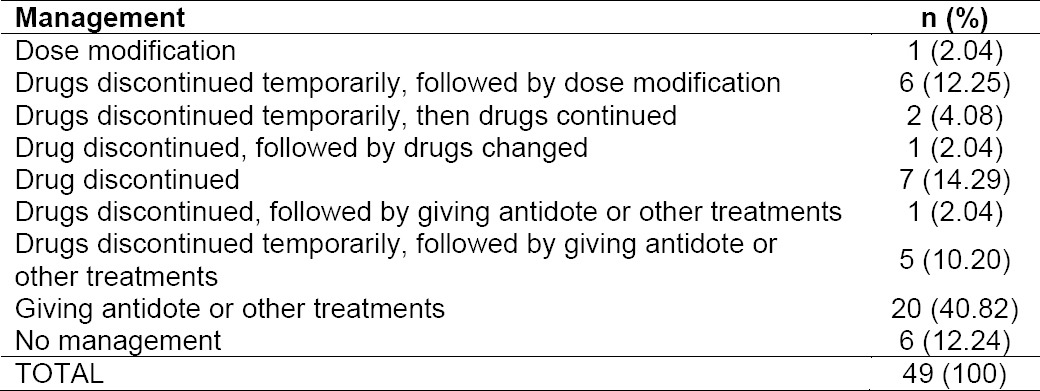
Profile of management of ADRs in CAD STEMI patients

The management of similar ADR from the same drug can be different. This can be determined by differences in the severity and the presence of other simultaneously occurring reactions. An example is in the case of UFH- and enoxaparin-induced hematuria, if gross hematuria does not occur, then the management is to decrease dose or discontinuation of drug temporarily. However, UFH-induced hematuria can cause complete discontinuation of the drug if the patient also experience thrombocytopenia due to UFH use (heparin-induced thrombocytopenia/HIT). In the case of a particular patient experiencing HIT, the patient may have increased APTT accompanied by hematuria so that the management is by stopping the UFH followed by the administration of other drug, fondaparinux. [17] demonstrated that if HIT occurred, heparin should be discontinued in patients with high and intermediate risk, and alternative anticoagulants should be initiated. Meanwhile, UFH-induced increase in APTT is generally managed by discontinuation of the drug temporarily, followed by dose modification. Some ADRs observed in the present study such as cough, increased levels of potassium and proteinuria were mostly not treated because the reactions were considered mild. Management of ADRs with level 3 severity involves administration of an antidote or other treatments. Drug discontinuation along with administration of antidote or other treatments were carried out upon the presence of hypotension and vomiting that occurred during fibrinolytic therapy with streptokinase. For hypotension, administration of atropine sulphate or fluid as sodium chloride or ringer lactate was carried out, while in the case of vomiting ondansetron treatment was initiated. Hypokalemia (potassium level in the blood <3.5 mEq/L) was treated by administration of potassium chloride (orally or injection), while heartburn caused by acetylsalicylic acid was treated with lansoprazole or omeprazole.

With regard to preventability, the most frequently occurring ADR; fell into the category “non-preventable” (79.59%). The criteria for determining the preventability of ADRs was based on the scale of Schumock-Thornton [18]. According to this scaling system, if the answer to any question set by the system (inappropriate dosage, route or frequency of drug, and drug interaction) is ‘yes’, then the ADRs are preventable. On the contrary, ‘no’ answer to each question will classify ADRs as non-preventable.

We observed significant effect from the number of drug types on the appearance of ADRs (p =0.001), Table 9. As seen in the table, hospitalization duration significantly determined ADRs experienced by patients (p = 0.011). Age is a risk factor for CAD that cannot be modified. This is in agreement with previous results showing confirmed that in male patients, the disease occurs at the age range of 50-65 years, while in female usually appears 10 years later following the menopause [3]. The present study did not show significant difference in the average age of patients who experienced ADRs and in those who did not (p = 0.957). The number of drug types used is also a risk factor for the occurrence of ADRs, as increasing the number, the potential for drug interactions followed, which may lead to hazardous effects on the patient.

**Tab. 9:**
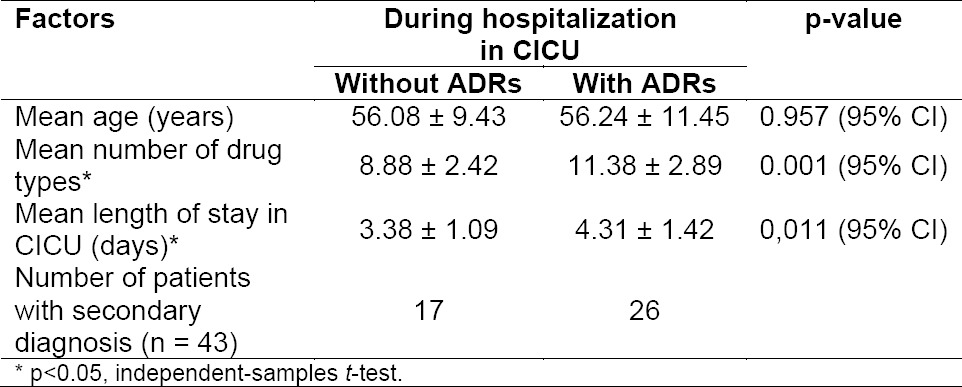
Profile of factors associated with ADRs

## Experimental

The study was performed in the Cardiac Intensive Care Unit (CICU) at a hospital in Bandung, during the period of December 2013 up to March 2014. This was an observational study which was done concurrently. Inclusion criteria were patients aged above 18 years old, male as well as female who were diagnosed of CAD STEMI. Meanwhile, the exclusion criteria were patients diagnosed with non-CAD STEMI, died within less than 48 hours after admission to CICU. The sources of data reviewed were patients’ medical records which included demographic data, the individual patient’s history (disease, medications, allergies, social, family), laboratory data, diagnosis, therapy, daily patient condition during treatment and at home, and length of stay at hospital; observation sheet which covered the individual patient’s vital signs, ECG, doctor’s instruction, therapy, and nurse’s daily record; medication profile including name of medication, dosage form, strength, route of administration, and duration of administration. Quantitative analysis conducted included patient demographic data, drug use, and the occurence of ADRs. ADRs were analyzed using a probability scale of Naranjo to assess the causal relationship of drug with ADRs. The severity and preventability of ADRs were analyzed by the scale of Hartwig and the scale of Schumock and Thornton, respectively. In addition, the analysis of ADRs management and the analysis of the factors determining ADRs occurrence were performed. Statistical analysis was conducted to compare the average of age, number of drug types, and duration of hospitalization among patients with and without ADRs. Significance in difference was assessed using independent-sample *t*-test.

## Conclusion

The present study revealed that organ systems most often affected by the ADRs were the cardiovascular system and the body electrolyte, followed by hematology and digestive systems. Further analyses demonstrated that most drugs probably caused ADRs, with the severity of the ADRs mostly fell into level 3/moderate. Moreover, the data unveiled that the average number of drug types and duration of hospitalization affected significantly the presence of ADRs. Taken together, most patients with CAD STEMI treated in CICU of the studied hospital experienced non-preventable ADRs and were treated with antidote or other treatments.

## Authors’ Statement

### Competing Interests

The authors declare no conflict of interest.
